# Desert ants avoid ambush predator pits depending on position, visual landmarks and trapped nestmates

**DOI:** 10.1098/rsos.241500

**Published:** 2024-12-18

**Authors:** Adi Bar, Eden Raveh-Pal, Aziz Subach, Susanne Foitzik, Inon Scharf

**Affiliations:** ^1^ School of Zoology, The George S. Wise Faculty of Life Sciences, Tel Aviv University, Tel Aviv, Israel; ^2^ Institute of Organismic and Molecular Evolution, Johannes Gutenberg University Mainz, Mainz, Rheinland-Pfalz, Germany

**Keywords:** group foraging, *Cataglyphis*, learning, pit-building predators, rescue behaviour, sociality

## Abstract

Central-place foragers face high predation risk when repeatedly using routes near their nest, as predators can learn to ambush them there. We investigated the factors influencing the likelihood of desert ant foragers falling into pitfall traps, simulating common predators such as antlions or spiders. We varied the spatial configuration of the pitfall traps, the presence of trapped nestmates and the availability of visual landmarks to study the workers’ susceptibility to falling into pits and their foraging success. Our results showed that a zigzag configuration of pits led to the fewest workers reaching the food reward, compared with straight-line and right-angled-triangle configurations. Over successive runs, workers became more efficient, with fewer falls into pits and faster food discovery, suggesting they either learned the locations of the pits or became more cautious. In any case, the ants learned about important landscape features close to their nest and the best way of navigating around it. Additionally, we examined the impact of trapped nestmates and visual landmarks. We found that pits marked with visual landmarks resulted in fewer falls. However, pits containing trapped nestmates attracted more ants, impaired food discovery and increased falling rates into pits, thereby imposing a twofold cost on ant foragers.

## 1. Introduction

Central-place foragers collect resources in their habitat and bring them back to a fixed, central home base [1–3]. Animals of different taxa use this foraging strategy, including social insects, like ants, honeybees and termites, nesting birds and burrowing rodents [4–6]. As central-place foragers need to return with the collected resources to their home base many of them have evolved sophisticated navigation skills and are capable of excellent spatial learning [6,7]. One example is path integration, which allows foragers to return directly to their home base even after using complex, indirect routes while foraging [5,8]. Another demonstration of cognitive skills is learning to avoid predators. Central-place foragers can learn to use less risky routes or patches while foraging or respond in other ways, such as pausing foraging when predators are detected [9–11].

Central-place foragers repeatedly use similar routes, like paths next to their nest or burrow, which make them more prone to attack by predators that exploit information on prey presence [12–14]. Ambush predators, or predators that do not chase after prey but attack from an ambush location, often choose routes or microhabitats that are heavily trafficked by prey [15,16]. As ambush predators rely on the prey coming to them, they encounter prey less frequently than their active predator relatives [17,18]. When prey fail to arrive, it would be better for such predators to relocate and search for another ambush location [19,20].

A complex question to answer is what dictates the encounter probabilities of ambush predators with prey. Clearly, ambushing in habitats with high prey abundance increases encounter probabilities. However, ambush predators may greatly differ in encounter rate based on locations owing to ‘shadow competition’, or the intercept of prey closer to its arrival source by other ambush predators [21,22]. Therefore, encounter rates do not only depend on the relative location of one ambush predator and its potential prey but also on other ambush predators nearby. In addition, some ambush predators lure the prey to approach them (i.e. ‘aggressive mimicry’), by emitting attractive visual, chemical or tactile signals [23,24]. An example is caudal lure by snakes, i.e. snakes that wave their tail that resembles arthropods, in order to attract prey [25,26].

Our goal here was to examine the effect of the spatial configuration of pitfall traps (hereafter, pits) and the cues they emit on the foraging behaviour of the desert ant, *Cataglyphis niger. Cataglyphis* ants forage individually without pheromone-based recruitment although they have some simple mechanism to recruit nestmates out of the nest to start foraging [27–29]. *Cataglyphis* foragers are skilled navigators utilizing multiple tools, such as landmarks, a panoramic view of the environment, path integration and distance estimation [30–33]. The spatial learning skills of *Cataglyphis* have been examined in the laboratory in different maze types (e.g. [34,35]). Foragers reach the food reward faster in successive runs in the same maze [35,36]. This improvement, however, is not only due to spatial learning but also increased motivation: foragers return with food and feed other workers in the nest and more workers consequently leave the nest to forage [36,37].

*Cataglyphis* species also rescue trapped nestmates, expressed mostly by pulling the trapped nestmate or digging around it [38–40]. The benefit of rescue behaviour is clear, i.e. helping a nestmate and maintaining a sufficient number of workers in the colony as the rearing cost of new workers is considerable. The costs are nevertheless more often assumed than demonstrated and can vary from energy and time loss, missed foraging opportunities, up to injury and death [39]. An exception is a study that examined the rescue of nestmates from spider webs and calculated the proportion of rescuers that got entangled in webs during web removal (approx. 6% of the workers; [41]). The pits utilized in our experiments imitate ambush predators, like pit-building antlions or ground spiders that hunt such ants in their natural habitat [42]. The distances moved by foraging ants may change with an increase in the number of pits, similar to the effect of increasing habitat complexity on the movement of other ants [43,44]. There are only two previous studies on desert ants and pitfall traps, which demonstrated that experienced workers fall less often into pits [42,45]. The study’s goal is to understand what the best ambush positioning strategies for ambush predators should be and what makes some pits more attractive than others. We were also interested in the cost of rescue if workers fall more often into pits in which a nestmate worker is already trapped.

We used different spatial configurations to examine the effect of ‘shadow competition’ among pits combined with the distance from the nest entrance, as the pit closer to the nest entrance may ‘shadow’ the ones farther away. We also examined the effect of two potential signals—an already-trapped nestmate inside the pit and a pit marked with a visual landmark, i.e. a plus sign (popping out from the pit’s centre approx. 1.5 cm above ground). We expected a decrease in the falling rate and food arrival time with experience or successive runs. We also expected to detect an effect of the spatial configuration according to shadow competition: foragers should more often fall into pits not ‘shadowed’ or obstructed by others. Finally, we expected that naive ants would be attracted to pits with an already-trapped nestmate but would be initially indifferent to the marked pits. On later runs, with accumulating experience, ants are expected to learn to avoid falling into pits with an already-trapped nestmate and to learn to avoid falling into pits marked with a visual landmark, as they are more conspicuous, and there is a better indication of their exact location. Trapped nestmates elicit rescue behaviour, and it could be that ant workers approaching the trapped nestmate in the pit would fall into it more frequently than into a control pit with no trapped nestmate. Ant behaviour, i.e. the tendency to fall into specific pits and/or to learn to avoid them, should also affect the decision of ambush predators of where to locate their ambush sites or whether to relocate them.

## 2. Materials and methods

### 2.1. General methods

**Figure 1.**
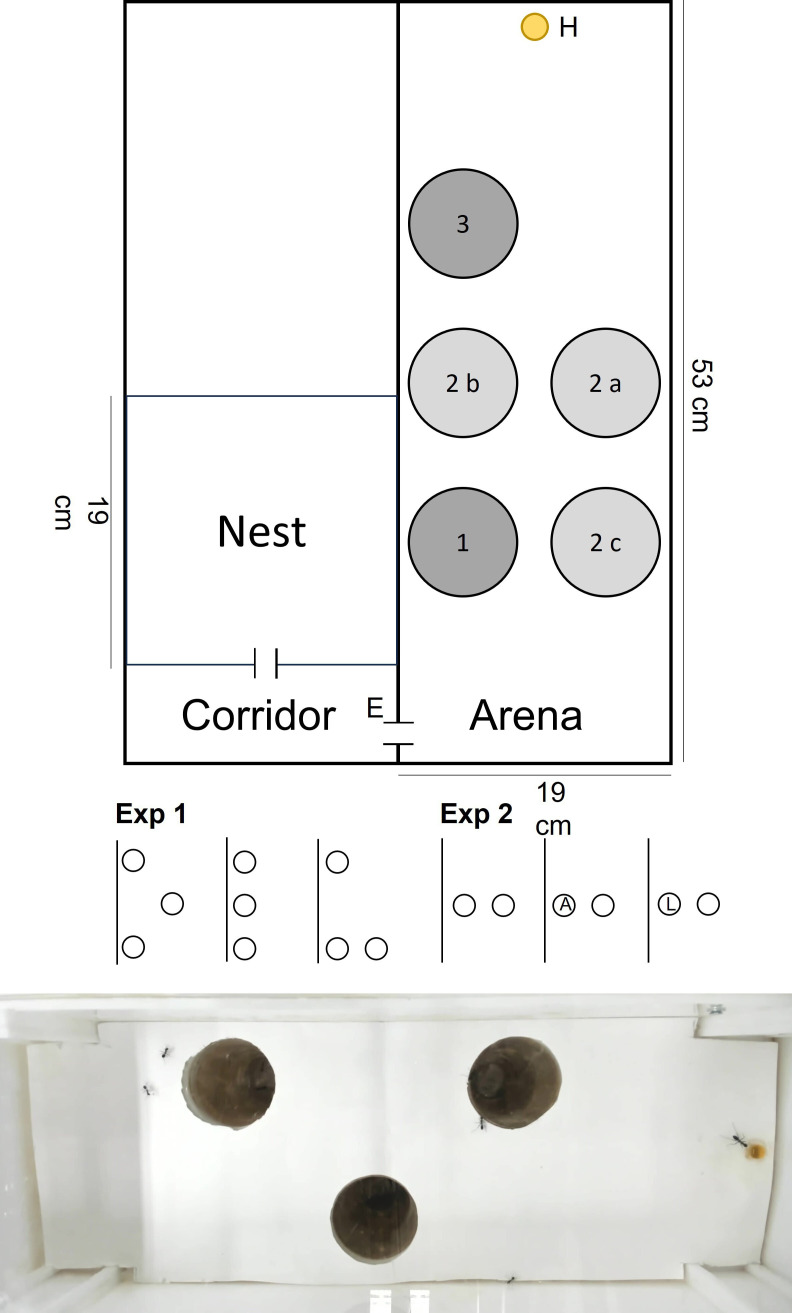
Nest and the experimental complex. The nest is connected to a corridor that is connected to the arena (E), which comprises three pits in Experiment 1 and two in Experiment 2. There were three pit configurations in Experiment 1: zigzag (pits in positions 1, 2a and 3), straight line (pits in positions 1, 2b and 3) and right-angled triangle (pits in positions 1, 2c and 3). In Experiment 2, the pits were always located in positions 2a and 2b. In the control, the two pits were identical. In the trapped nestmate treatment (A), we placed an ant either in position 2a or 2b. In the marked pit treatment (L), a plus sign was placed either in position 2a or 2b. H stands for the food reward (0.5 g honey). Five workers are present in the arena in the photo below, of which one reaches the food reward (right).

*Cataglyphis niger* colonies were collected in Tel Baruch sand dunes (32.132° N, 34.788° E), a small enclave (1.5 × 0.5 km) of a dune habitat in northwestern Tel Aviv. Each colony was kept in the laboratory for at least one week before taking part in the experiment. Each colony’s box contained two parts: a darkened nest with two water tubes and a forging arena exposed to external light. The colony boxes were kept on a shaded balcony and exposed to natural thermal conditions. Once per week, the colony received food, several *Tenebrio molitor* larvae and fresh water. We conducted two experiments; in each, 10 different colonies took part. A week prior to the experiment, 120 ants per colony were separated into three groups of 40 ants with no queen or brood. Each such group was kept in an acclimatization box (the nest; 19 × 19 × 10 cm; l × w × h; figure 1) with access to water but without food in the experimental room with a regime of 12 h : 12 h light : dark and 28°C. Right before the experiment, the acclimatization box was connected to the experimental box, which included a test arena (53 × 19 cm) and a corridor (4 × 19 cm) connecting the test arena to the acclimatization box. In the arena, there were five pits, which could be exposed or covered, according to the treatment. We placed a food reward (0.5 g honey) at the arena’s end (figure 1).

Each of the three different groups of 40 workers was allocated to a single treatment. All workers in both experiments were allowed to search in the arena (but only some decided to do so) for three successive runs with an interval of 30 min in between. Each run lasted 50 min, or 10 min from the arrival of the first ant worker at the food reward, the shortest of the two. Ant workers that fell into the pits were covered with sand for 2 min as a negative feedback. Between runs, all the ants from the arena and pitfalls were collected and returned to the acclimatization box. All runs were filmed. We obtained the following data from the video: number of workers falling into each pit, number of workers arriving at the food reward, arrival time of the first worker at the food reward, falling time of the first worker into a pit, movement distance of the first worker arriving at the food reward and the movement distance of the first worker falling into a pit. In order to calculate movement distances, we cut the films into segments, split each into frames (every second) and monitored the ant location using ImageJ [46]. It was impossible to use blinded methods because the treatments were clear to the observers.

### 2.2. Experiment 1: influence of spatial pit configuration on ant foraging

We examined the effect of the spatial configuration of pitfalls on the foraging behaviour of the desert ants. All three treatments comprised three exposed pits but in a different spatial configuration (figure 1): (i) a zigzag configuration: two pits along the wall adjacent to the nest entrance and one in the middle along the opposite wall; (ii) a straight line: all pits were adjacent and parallel to the entrance wall but in an increasing distance from the entrance; and (iii) a right-angled-triangle configuration: two pits close to the nest entrance and one far away. We chose these three spatial configurations because they differed in ‘shadow competition’, or the effect that the pit closest to the nest entrance had on pits downstream, and because pits 1 and 3 remained fixed in their location and pit 2, in contrast, was the only one to change location (figure 1).

#### 2.2.1. Statistical analysis

We used linear mixed models with a normal error distribution to analyse the effect of treatment, run number and their interaction on each of the response variables: number of workers arriving at the food reward, time until the first worker arrived at the food reward and the distance it travelled (only for those that arrived), number of workers falling into pits, number of workers falling into each specific pit, time from the entry to the arena until the first worker fell into a pit and the distance it travelled. When the interaction was not significant, it was removed and the test was redone. Colony ID was referred to as a random variable. In some cases (see §3), the response variables were not normally distributed, and we used either a square root or a log_10_ transformation, the best of the two bringing the distribution closer to a normal one. When none of the workers reached the food reward or fell into pits, the replication received 0 for the number of workers and was omitted from the analysis of arrival or falling times. We used the zigzag configuration treatment and the marked pit treatment as references in the first and second experiments, respectively. We also compared specific pits (those in locations 1, 2 and 3; figure 1) among treatments using a similar test. We used Systat v. 13 (Systat Software, Inc., San Jose, CA, USA) for the statistical analyses.

### 2.3. Experiment 2: influence of cues associated with pits on ant foraging

We examined how ants respond to a pit with a nestmate inside versus a control with an empty pit, and a pit marked with a visual landmark versus a control, an empty pit. There were always two pits exposed in the middle of the arena. We applied three treatments: (i) a control, or two empty pits; (ii) one pit with a free, untied nestmate inside, which could not climb out, placed a minute before each run, and another empty pit; and (iii) one pit marked with a plus sign, placed on a narrow plastic attached to the pit bottom and popping out approximately 1.5 cm above ground, and another empty pit (figure 1, Experiment 2). The location of the two different pits in the two latter treatments was the same during the three successive runs but differed among colonies (randomly chosen). For simplicity, only one configuration is presented in figure 1 (Experiment 2).

#### 2.3.1. Statistical analysis

Like in Experiment 1, we used linear mixed models to analyse the effect of treatment (trapped nestmate treatment, marked pit treatment and a control), run number (1, 2 and 3) and their interaction on each of the response variables: number of workers arriving at the food reward, time until the first worker arrived at the food reward and the distance it travelled, number of workers falling into pits, number of workers falling into each specific pit, time from the entry to the arena until the first worker fell into a pit, and the distance it travelled. Next, we conducted a complementary analysis. Because the trapped nestmate treatment and marked pit treatment each comprised two different pits, one either marked with a visual landmark or a trapped nestmate and control (an empty pit), it was possible to directly compare within each treatment the number of workers falling into each of the two pits. We subtracted the number of workers falling into the control, the empty unmarked pit, from the treatment pit (marked or trapped nestmate). If this difference is positive, more workers fall into the treatment pit whereas if it is negative more workers fall into the control pit. We examined whether this difference was affected by treatment, run number and their interaction using a linear mixed model.

## 3. Results

### 3.1. Experiment 1: influence of spatial pit configuration on ant foraging

**Figure 2.**
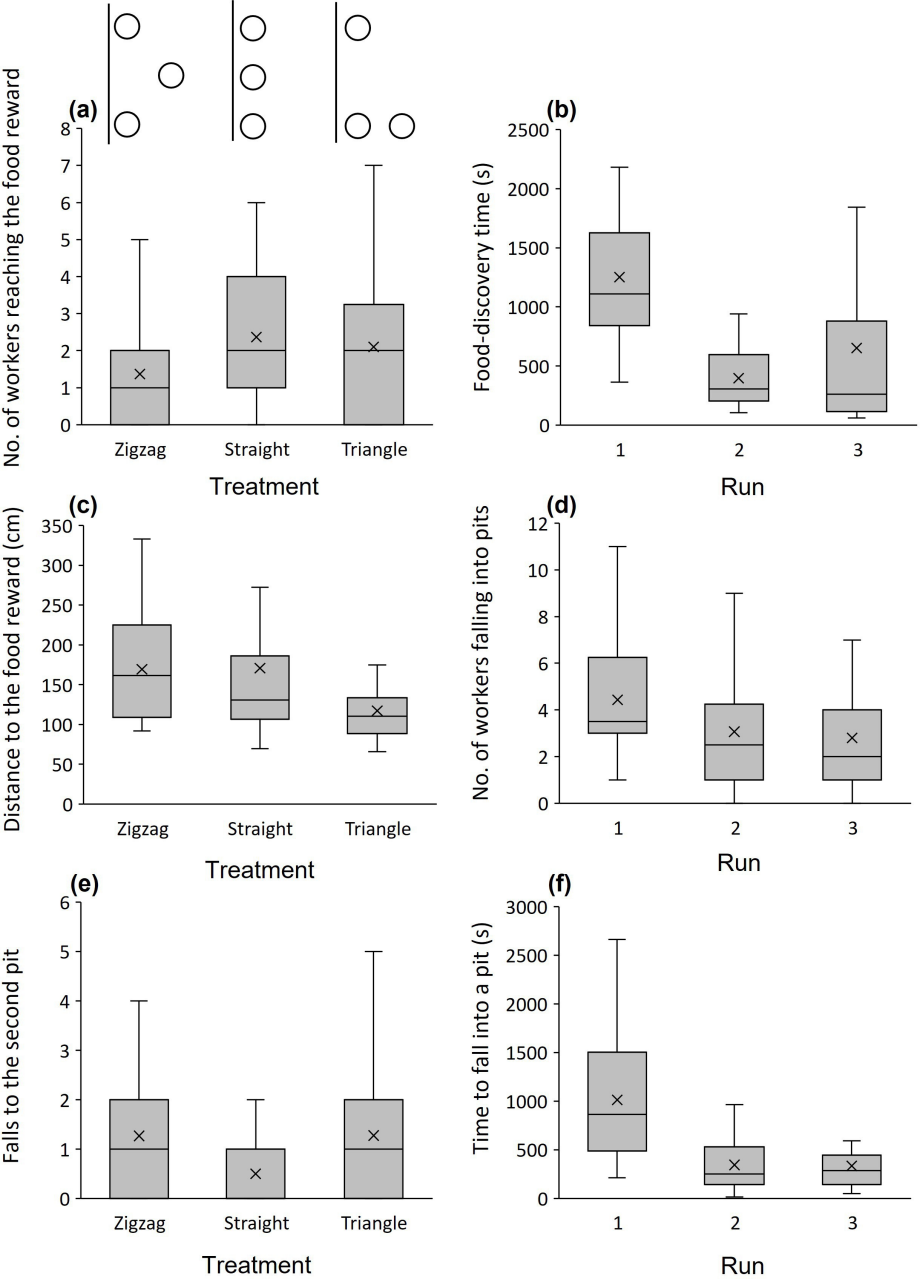
Results of Experiment 1: influence of spatial pit configuration on ant foraging. The effect of treatment and/or run on (*a*) the number of workers arriving at the food reward (zigzag < straight line and right-angle triangle), (*b*) food-discovery time by the first worker (decreasing with runs), (*c*) distance of that worker to the food reward (zigzag > right-angle triangle), (*d*) number of workers falling into pits (decreasing with runs), (*e*) number of workers falling into the second pit (zigzag > straight line; see figure 1) and (*f*) the time until the first worker fell into a pit (faster with runs). Medians (horizontal lines), means (×), 25%–75% quartiles (boxes) and all data without outliers (whiskers) are presented. Some of the analysis was done on transformed response variables.

The lowest number of workers arrived at the food reward when pits were arranged in a zigzag configuration and the highest when pits were arranged in a straight line (log_10_-transformed; zigzag versus straight: *t* = 2.948, *p* = 0.004, zigzag versus right-angled triangle: *t* = 1.994, *p* = 0.049; figure 2*a*). Run number had no effect (*t* = 0.367, *p* = 0.715) as well as the two-way treatment × run interaction (*p* > 0.639). Arrival times of workers arriving at the food reward were not affected by treatment (log_10_-transformed; zigzag versus straight and zigzag versus right-angled triangle: *t* = −0.074 and −0.116, *p* = 0.941 and 0.908) but decreased with successive runs (*t* = −4.480, *p* < 0.001; figure 2*b*). The treatment × run interaction was not significant (*p* > 0.143). Distances moved by the first worker to reach the food reward were greater in the zigzag configuration than in the right-angled-triangle configuration (square-root transformed; *t* = −2.353, *p* = 0.023; figure 2*c*) but did not differ between the zigzag and straight-line configurations (*t* = −0.283, *p* = 0.778). The run number was not significant (*t* = −0.868, *p* = 0.389) as well as the treatment × run interaction (−0.393 < *t* < 0.014, *p* > 0.695). Regarding the workers falling into pits, more workers fell into pits in the first run than later (square-root transformed; *t* = −2.735, *p* = 0.008; figure 2*d*). There was no difference among treatments (zigzag versus straight and zigzag versus right-angled triangle: *t* = −0.754 and −0.384, *p* = 0.453 and 0.702) nor was the treatment × run interaction significant (*p* > 0.388). Although the number of workers falling was similar across treatments, the number of workers falling into the second pit differed among treatments: more workers fell into the second pit in the zigzag treatment than in the straight-line treatment (*t* = −2.610, *p* = 0.011; figure 2*e*) with a similar number of falls in the right-angled-triangle treatment to the zigzag treatment (*t* = −0.043, *p* = 0.966). Run number had no effect (*t* = 0.420, *p* = 0.676) as well as the treatment × run interaction (*p* > 0.483). Regarding the first pit, here there was no difference among treatments (zigzag versus straight and zigzag versus right-angled triangle: *t* = 0.555 and −0.005, *p* = 0.580 and 0.996), but fewer workers fell into this pit with successive runs (*t* = −3.862, *p* < 0.001). The treatment × run interaction was not significant (*p* > 0.393). Last, there was no effect of any treatment, run number or treatment × run interaction on the number of workers falling into the third pit (*p* > 0.173 for all). Workers required less time to fall into pits in the second and third runs than in the first run (log_10_-transformed; *t* = −5.132, *p* < 0.001; figure 2*f*). There were no differences among treatments (zigzag versus straight and zigzag versus right-angled triangle: *t* = −1.838 and −1.271, *p* = 0.071 and 0.208). The treatment × run interaction was not significant too (*p* > 0.513). The distances travelled by the first worker to fall into a pit were similar across the treatments of spatial configuration and runs (treatment: square-root transformed; −0.351 < *t* < 0.412, *p* > 0.681; run: *t* = −0.103, *p* = 0.918; the treatment × run interaction: *t* < 0.890, *p* > 0.377). See the electronic supplementary material, table S1 for a summary table of this experiment per treatment per run.

### 3.2. Experiment 2: influence of cues associated with pits on ant foraging

#### 3.2.1. Between-treatment comparisons

**Figure 3.**
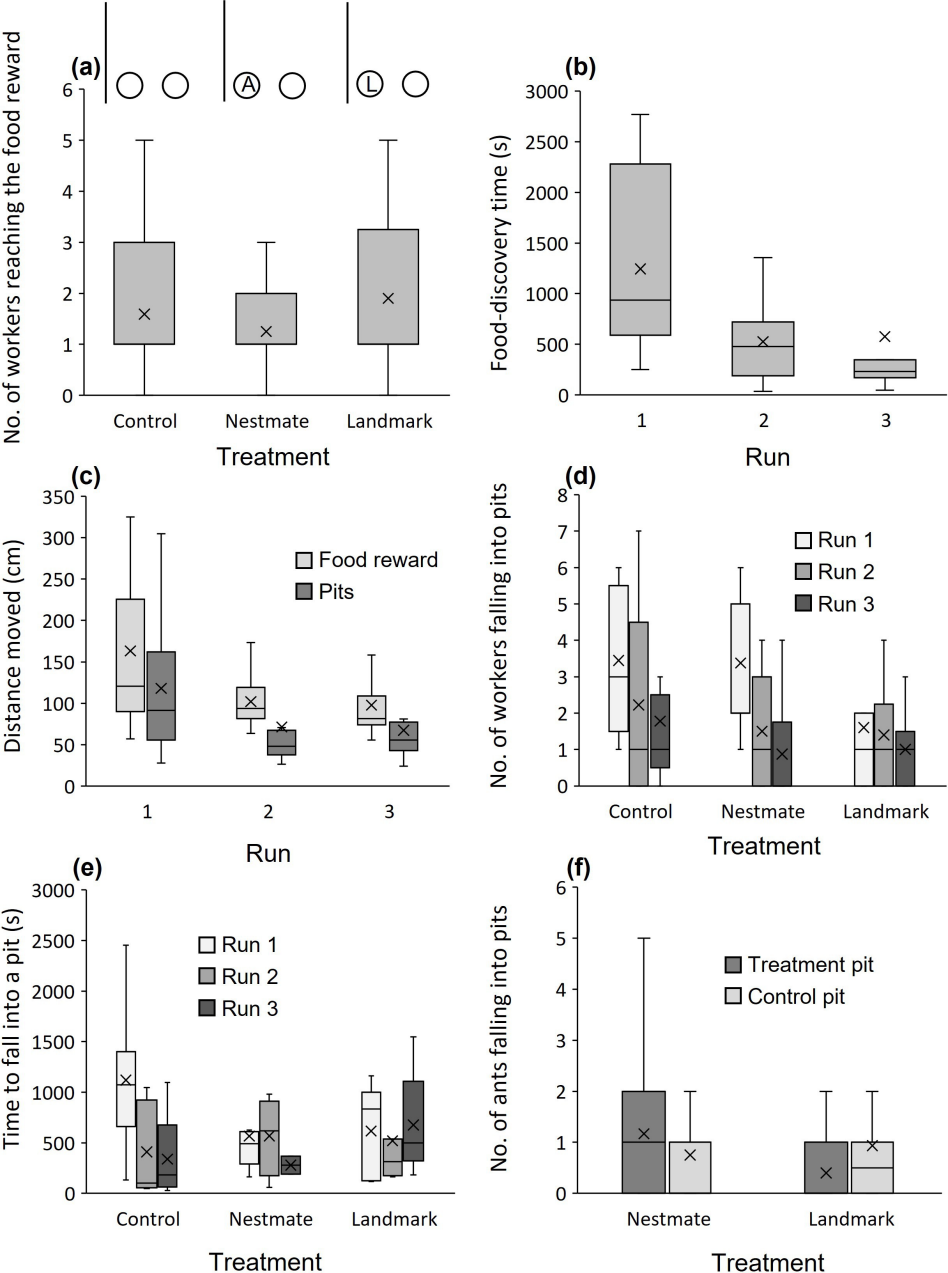
Results of Experiment 2: influence of cues associated with pits on ant foraging. The effect of treatment and/or run number on (*a*) the number of workers arriving at the food reward (marked pit treatment > trapped nestmate treatment), (*b*) food-discovery time by the first worker (faster with runs), (*c*) distance moved by the first worker discovering the food or falling into a pit (decreasing with runs), (*d*) number of workers falling into pits (fewer with runs except for the marked pit treatment), (*e*) time until the first worker fell into a pit (longer in the first run of the control and then reaching a similar level to all other treatments and runs) and (*f*) falls into pits, separately presented for each pit in the trapped nestmate and marked pit treatments versus the control pit (more workers fell into the pit with a trapped nestmate than into the control pit and more workers fell into the control pit than into the marked pit). Medians (horizontal lines), means (×), 25%–75% quartiles (boxes) and all data without outliers (whiskers) are presented. Some of the analysis was done on transformed response variables.

More workers arrived at the food in the marked pit treatment than in the trapped nestmate treatment (square-root transformed; *t* = −2.084, *p* = 0.041; figure 3*a*), with no difference between the marked pit treatment and control (*t* = −0.709, *p* = 0.481). Run number had no effect (*t* = −0.267, *p* = 0.791) as well as the treatment × run interaction (*p* > 0.169). There is no difference in arrival times at the food reward between treatments (log_10_-transformed; marked pit treatment versus trapped nestmate treatment and marked pit treatment versus the control: *t* = −0.884 and −0.427, *p* = 0.381 and 0.671), but first arrivals were faster in the second and third runs than in the first run (*t* = −4.227, *p* < 0.001; figure 3*b*). The treatment × run interaction was not significant (*p* > 0.236). The distances moved by the first worker to reach the food reward did not differ between treatments (log_10_-transformed; marked pit treatment versus trapped nestmate treatment and marked pit treatment versus the control: *t* = −0.557 and 1.015, *p* = 0.580 and 0.314) but decreased with runs, mostly between the first and second runs (*t* = −3.238, *p* = 0.002; figure 3*c*). The treatment × run interaction was not significant (*p* > 0.455). The number of workers falling into pits was affected by an interaction of treatment and run (square-root transformed; marked pit versus trapped nestmate: *t* = −2.237, *p* = 0.029): workers fell less frequently into pits in successive runs, except for the marked pit treatment. In this case, most workers avoided the pit already from the first run, with no further improvement (figure 3*d*). Considering the main effects of the above-described test (number of workers falling into pits), the run was not significant (*t* = −0.324, *p* = 0.747) but treatment was (marked pit versus trapped nestmate: *t* = 2.526, *p* = 0.014; marked pit versus control: *t* = 2.132, *p* = 0.037). The time until workers fell into a pit was affected by the interaction of treatment and run (log_10_-transformed; marked pit versus control: *t* = −2.737, *p* = 0.009; figure 3*e*): it was especially longer in the control in the first run but then reached a similar level to all other treatments and runs. Considering the main effects of this test, run number as a main effect was not significant (*t* = 0.344, *p* = 0.733) but the marked pit treatment differed from the control (*t* = 2.181, *p* = 0.035), whereas it did not differ from the trapped nestmate treatment (*t* = 0.687, *p* = 0.497). Distances moved by the first worker to fall into a pit were shorter in the second and third runs than in the first run (log_10_-transformed; *t* = −2.894, *p* = 0.006; figure 3*c*). Neither treatment (marked pit treatment versus trapped nestmate treatment and marked pit treatment versus the control: *t* = −1.372 and 0.539, *p* = 0.178 and 0.593) nor the treatment × run interaction was significant (*p* > 0.836).

#### 3.2.2. Within-treatment comparisons

We focused next on the marked pit and trapped nestmate treatments. The difference in the number of workers falling (number of workers falling into the control, empty unmarked pit subtracted from the treatment pit, i.e. the marked pit or the pit with a trapped nestmate) differed between treatments (*t* = 2.084, *p* = 0.043): more workers fell into the pit with a trapped nestmate than into the control pit and more workers fell into the control pit than into the marked pit (figure 3*f*). Run was not significant (*t* = −1.202, *p* = 0.236) as well as the treatment × run interaction (*t* = −1.783, *p* = 0.082). See the electronic supplementary material, table S1 for a summary table of this experiment per treatment per run.

## 4. Discussion

Our two experiments on the influence of pit configuration, trapped nestmates or visual landmarks on foraging success and falling probability into pits have two important outcomes. First, pit configuration, even when the number of pits is identical, can greatly affect food discovery. Here, the worst spatial configuration from the ant perspective was a zigzag and the best was a straight line. Such configurations can also affect the distance that ant workers must travel to find food. Second, foraging ant workers have a higher likelihood of falling into pits with a trapped nestmate, which indicates they are somehow attracted to it. This result is meaningful as an indication of the cost of sociality—getting trapped while trying to rescue nestmates. However, ant workers fall less frequently into such pits with successive runs, suggesting a learning process. In addition, marking a pit with a visual landmark reduced the number of workers falling into it. The reason for this response is unclear, but the visual landmark might have made the pits more visible to the ants.

The configuration of pits greatly affected the probability of falling into pits. Specifically, the zigzag configuration impaired the arrival of workers at the food reward, which was also reflected in later arrival. The reason is perhaps that it is more difficult to bypass such a configuration. Studies in landscape or movement ecology usually increase habitat complexity by increasing the habitat area covered by obstacles. Increasing complexity clearly impairs movement [43,44,47], but the configuration plays a role too, as shown here. Similarly, McIntyre & Wiens [48] demonstrated in ground darkling beetles that obstacles of different sizes, even when the overall cover remains constant, differed in their effect on several movement characteristics. For example, the number of stops followed a hump-shaped pattern with obstacle size. Furthermore, the spatial distribution pattern of food interacted with hunger level to affect movement in a congeneric darkling beetle [49]. Our experiment provides further evidence for the importance of the spatial configuration of pits. Fewer ants fell into the second pit in the straight-line treatment than into the second pit in the two other treatments. The reason was perhaps that taking the nest entrance into consideration, falling into the second pit in the straight-line configuration required the sharpest turn of all treatments (figure 1). This is also relevant from the ambush predator’s perspective, as a straight-line configuration is more prone to shadow competition than other ones. As predators farther away from the nest entrance, especially in this configuration, encounter fewer prey, they should eventually relocate and choose a better ambush location.

Two other more general results that emerge are the improvement with successive runs in the arena, but mostly between the first and second runs with a much more moderate improvement between the second and third runs. Faster arrival at the food is caused either by a shorter movement to the food reward with runs (Experiment 2) or by a faster entry to the maze (Experiment 1; not shown), and it is also evident when ants of the same species search in a maze [35,36]. Ants also fall faster into the pits although in lower numbers than in the first run, probably because they entered the arena faster in the second and third runs or because they moved faster. The decrease in the number of workers falling into pits (mainly into the first one) agrees with a previous study in the same species and similar arenas with pits [42]. Both reflect a learning process, which is a mixture of spatial learning, i.e. shorter tracks to reach food, and associative learning or elevated motivation to search in the arena, i.e. faster entry to the arena when the door is opened.

The type of pit, i.e. whether it was marked with a visual landmark or contained a trapped nestmate, affected not only the number of workers falling into the pit but also the number of workers arriving at the food reward. When a nestmate was trapped in one of the pits, fewer workers arrived at the food and more workers fell into that pit. Many studies emphasized the positive contribution of sociality to group members. For example, group members can detect new food resources faster than individual foragers [50,51], and groups are often more protected against predators [52–54]. However, we demonstrated here a potential twofold cost of sociality and specifically of rescue behaviour, which was often neglected in similar studies. First, trapped nestmates attract other workers, probably for the sake of rescue, which commonly takes place in this and other congeneric species [38,40]. Such attracted workers fall into the same pit and are exposed to predation risk. Similar results were obtained when ants rescue nestmates from spider webs or antlions and get themselves entangled in such webs or fall into such pits [41,55]. If prey arrives faster, ambush predators can accelerate their handling of prey, increase consumption rate, dump the old prey item earlier and move to the fresh one or even simultaneously handle more than a single prey individual [56–59]. Second, the delayed discovery of food is another potential cost of rescue behaviour and is probably caused by the higher number of workers in this treatment falling into pits. Delayed arrival at the food might be important in this species in particular, because *Cataglyphis* forages for scattered (mostly dead) prey items [60,61], and delayed arrival might mean that foragers of another colony or species have already removed the prey. Foraging costs have been shown in the rescue behaviour of rats when rescuing is prioritized over high-quality food [62].

Marked pits were more likely to be avoided by workers and they fell into marked pits in lower numbers. Interestingly, the difference was mostly evident in the first run: whereas workers learned not to fall into the pits in other treatments, they fell less frequently to marked pits from the beginning. It could be that when the pit is marked, workers approached it less often, and instead used it as a ‘landmark’ to navigate in the arena. *Cataglyphis* ants often use landmarks during their navigation [33,63]. This result also highlights the importance of being conspicuous for ambush predators because they are approached by prey and rely on not being detected [64]. Our results are meaningful for ambush site selection by ambush predators as the pit location relative to other pits and to the nest entrance affects prey arrivals. Most ambush predators respond to prey, i.e. either choose sites rich with prey or relocate fast in the absence of prey capture [9,15,65]. Some, however, do not choose sites especially rich with prey and/or require a long time to relocate when prey cease to arrive [66,67]. A future study can mark antlion pits in the field versus a control of unmarked pits and examine how it affects the likelihood of ants falling into the marked versus unmarked pits.

In summary, our study has implications for foraging ants and their ambush predators. Regarding ants, it demonstrates the costs of rescue behaviour and how they are differently affected by the spatial configuration of pitfall traps or ambush locations of predators near their nest. Ambush predators experience varying success depending on their location relative to competitors and the prey nest. Future studies should perhaps use real predators (spiders or antlions) and examine this predator–prey interaction for longer, allowing each player to adjust its behaviour. Another interesting direction would be to conduct similar experiments in ant species that rely on recruitment and examine whether environmental cues, pitfall traps and their spatial orientation have qualitatively similar effects on ant species of different foraging strategies.

## Data Availability

The dataset is available online at FigShare: https://doi.org/10.6084/m9.figshare.27958395.v1. Supplementary material is available online [68].

## References

[1] Davidson DW. 1977 Foraging ecology and community organization in desert seed‐eating ants. Ecology **58**, 725–737. (10.2307/1936209)

[2] Orians GH, Pearson NE. 1979 On the theory of central place foraging. In Analysis of ecological systems (eds DJ Horn, RD Mitchell, GR Stairs), pp. 154–177. Columbus, OH: Ohio State University Press.

[3] Bell WJ. 1990 Central place foraging. In Searching behaviour: the behavioural ecology of finding resources, pp. 171–187. Dordrecht, The Netherlands: Springer. (10.1007/978-94-011-3098-1_12)

[4] Kacelnik A. 1984 Central place foraging in starlings (Sturnus vulgaris). I. Patch residence time. J. Anim. Ecol. **53**, 283. (10.2307/4357)

[5] Dyer FC. 1998 Spatial cognition: lessons from central-place foraging insects. In Animal cognition in nature (eds RP Balda, IM Pepperberg, AC Kamil), pp. 119–154. San Diego, CA: Academic Press. (10.1016/B978-012077030-4/50057-X)

[6] Collett M, Collett TS. 2007 Spatial aspects of foraging in ants and bees. In Invertebrate neurobiology (eds C North, RJ Greenspan), pp. 467–502. Cold Spring Harbor, NY: Cold Spring Harbor Laboratory Press.(Cold Spring Harbor Monograph Series 49).

[7] Iorio-Merlo V, Graham IM, Hewitt RC, Aarts G, Pirotta E, Hastie GD, Thompson PM. 2022 Prey encounters and spatial memory influence use of foraging patches in a marine central place forager. Proc. R. Soc. B **289**, 20212261. (10.1098/rspb.2021.2261) PMC8889173 35232237

[8] Müller M, Wehner R. 1994 The hidden spiral: systematic search and path integration in desert ants, Cataglyphis fortis. J. Comp. Physiol. A **175**, 525–530. (10.1007/BF00199474)

[9] MacKay WP. 1982 The effect of predation of western widow spiders (Araneae: Theridiidae) on harvester ants (Hymenoptera: Formicidae). Oecologia **53**, 406–411. (10.1007/BF00389022) 28311749

[10] Huntingford FA, Wright PJ. 1989 How sticklebacks learn to avoid dangerous feeding patches. Behav. Processes **19**, 181–189. (10.1016/0376-6357(89)90040-5) 24895911

[11] Ings TC, Chittka L. 2009 Predator crypsis enhances behaviourally mediated indirect effects on plants by altering bumblebee foraging preferences. Proc. R. Soc. B **276**, 2031–2036. (10.1098/rspb.2008.1748) PMC2677241 19324797

[12] Dukas R, Morse DH. 2003 Crab spiders affect flower visitation by bees. Oikos **101**, 157–163. (10.1034/j.1600-0706.2003.12143.x)

[13] Elizalde L, Farji‐brener A. 2012 To be or not to be faithful: flexible fidelity to foraging trails in the leaf‐cutting ant Acromyrmex lobicornis. Ecol. Entomol. **37**, 370–376. (10.1111/j.1365-2311.2012.01368.x)

[14] Gable TD, Johnson-Bice SM, Homkes AT, Fieberg J, Bump JK. 2023 Wolves alter the trajectory of forests by shaping the central place foraging behaviour of an ecosystem engineer. Proc. R. Soc. B **290**, 20231377. (10.1098/rspb.2023.1377) PMC10645084 37935367

[15] Harwood JD, Sunderland KD, Symondson WOC. 2003 Web‐location by linyphiid spiders: prey‐specific aggregation and foraging strategies. J. Anim. Ecol. **72**, 745–756. (10.1046/j.1365-2656.2003.00746.x)

[16] Clark RW. 2004 Timber rattlesnakes (Crotalus horridus) use chemical cues to select ambush sites. J. Chem. Ecol. **30**, 607–617. (10.1023/b:joec.0000018632.27010.1e) 15139311

[17] Huey RB, Pianka ER. 1981 Ecological consequences of foraging mode. Ecology **62**, 991–999. (10.2307/1936998)

[18] Werner EE, Anholt BR. 1993 Ecological consequences of the trade-off between growth and mortality rates mediated by foraging activity. Am. Nat. **142**, 242–272. (10.1086/285537) 19425978

[19] Hauber ME. 1999 Variation in pit size of antlion (Myrmeleon carolinus) larvae: the importance of pit construction. Physiol. Entomol. **24**, 37–40. (10.1046/j.1365-3032.1999.00109.x)

[20] Nakata K, Ushimaru A. 1999 Feeding experience affects web relocation and investment in web threads in an orb-web spider, Cyclosa argenteoalba. Anim. Behav. **57**, 1251–1255. (10.1006/anbe.1999.1105) 10373258

[21] Lubin Y, Henschel JR, Baker MB. 2001 Costs of aggregation: shadow competition in a sit‐and‐wait predator. Oikos **95**, 59–68. (10.1034/j.1600-0706.2001.950107.x)

[22] Scharf I, Ruxton GD. 2023 Shadow competition: its definition, prevalence, causes and measurement. Oikos **2023**, e09774. (10.1111/oik.09774)

[23] Wignall AE, Taylor PW. 2011 Assassin bug uses aggressive mimicry to lure spider prey. Proc. R. Soc. B **278**, 1427–1433. (10.1098/rspb.2010.2060) PMC3061146 20980305

[24] Jackson RR, Cross FR. 2013 A cognitive perspective on aggressive mimicry. J. Zool. **290**, 161–171. (10.1111/jzo.12036) PMC3748996 23976823

[25] Reiserer RS, Schuett GW. 2008 Aggressive mimicry in neonates of the sidewinder rattlesnake, Crotalus cerastes (Serpentes: Viperidae): stimulus control and visual perception of prey luring. Biol. J. Linnean Soc. **95**, 81–91. (10.1111/j.1095-8312.2008.01016.x)

[26] Glaudas X, Alexander GJ. 2017 A lure at both ends: aggressive visual mimicry signals and prey-specific luring behaviour in an ambush-foraging snake. Behav. Ecol. Sociobiol. **71**, 2. (10.1007/s00265-016-2244-6)

[27] Amor F, Ortega P, Cerdá X, Boulay R. 2010 Cooperative prey-retrieving in the ant Cataglyphis floricola: an unusual short-distance recruitment. Insectes Soc. **57**, 91–94. (10.1007/s00040-009-0053-x)

[28] Razin N, Eckmann JP, Feinerman O. 2013 Desert ants achieve reliable recruitment across noisy interactions. J. R. Soc. Interface **10**, 20130079. (10.1098/rsif.2013.0079) 23486172 PMC3627090

[29] Boulay R, Aron S, Cerdá X, Doums C, Graham P, Hefetz A, Monnin T. 2017 Social life in arid environments: the case study of Cataglyphis ants. Annu. Rev. Entomol. **62**, 305–321. (10.1146/annurev-ento-031616-034941) 27860520

[30] Wehner R. 2003 Desert ant navigation: how miniature brains solve complex tasks. J. Comp. Physiol. A **189**, 579–588. (10.1007/s00359-003-0431-1) 12879352

[31] Wehner R. 2009 The architecture of the desert ant’s navigational toolkit (Hymenoptera: Formicidae). Myrmecol. News **12**, 85–96.

[32] Collett M. 2012 How navigational guidance systems are combined in a desert ant. Curr. Biol. **22**, 927–932. (10.1016/j.cub.2012.03.049) 22521785

[33] Fleischmann PN, Rössler W, Wehner R. 2018 Early foraging life: spatial and temporal aspects of landmark learning in the ant Cataglyphis noda. J. Comp. Physiol. A **204**, 579–592. (10.1007/s00359-018-1260-6) PMC5966506 29679143

[34] Chameron S, Schatz B, Pastergue-Ruiz I, Beugnon G, Collett TS. 1998 The learning of a sequence of visual patterns by the ant Cataglyphis cursor. Proc. R. Soc. Lond. B. **265**, 2309–2313. (10.1098/rspb.1998.0576)

[35] Saar M, Gilad T, Kilon-Kallner T, Rosenfeld A, Subach A, Scharf I. 2017 The interplay between maze complexity, colony size, learning and memory in ants while solving a maze: a test at the colony level. PLoS One **12**, e0183753. (10.1371/journal.pone.0183753) 28837675 PMC5570381

[36] Bega D, Samocha Y, Yitzhak N, Saar M, Subach A, Scharf I. 2020 Non-spatial information on the presence of food elevates search intensity in ant workers, leading to faster maze solving in a process parallel to spatial learning. PLoS One **15**, e0229709. (10.1371/journal.pone.0229709) 32109253 PMC7048290

[37] Gilad T, Dorfman A, Subach A, Libbrecht R, Foitzik S, Scharf I. 2022 Evidence for the effect of brief exposure to food, but not learning interference, on maze solving in desert ants. Integr. Zool. **17**, 704–714. (10.1111/1749-4877.12622) 34958517

[38] Nowbahari E, Scohier A, Durand JL, Hollis KL. 2009 Ants, Cataglyphis cursor, use precisely directed rescue behavior to free entrapped relatives. PLoS One **4**, e6573. (10.1371/journal.pone.0006573) 19672292 PMC2719796

[39] Miler K, Turza F. 2021 ‘O sister, where art thou?’—a review on rescue of imperiled individuals in ants. Biology **10**, 1079. (10.3390/biology10111079) 34827072 PMC8614747

[40] Bar A, Gilad T, Massad D, Ferber A, Ben-Ezra D, Segal D, Foitzik S, Scharf I. 2023 Foraging is prioritized over nestmate rescue in desert ants and pupae are rescued more than adults. Behav. Ecol. **34**, 1087–1096. (10.1093/beheco/arad083)

[41] Kwapich CL, Hölldobler B. 2019 Destruction of spiderwebs and rescue of ensnared nestmates by a granivorous desert ant (Veromessor pergandei). Am. Nat. **194**, 395–404. (10.1086/704338) 31553216

[42] Bar A, Marom C, Zorin N, Gilad T, Subach A, Foitzik S, Scharf I. 2022 Desert ants learn to avoid pitfall traps while foraging. Biology **11**, 897. (10.3390/biology11060897) 35741418 PMC9219994

[43] Gibb H, Parr CL. 2010 How does habitat complexity affect ant foraging success? A test using functional measures on three continents. Oecologia **164**, 1061–1073. (10.1007/s00442-010-1703-4) 20589395

[44] Radnan GN, Gibb H, Eldridge DJ. 2018 Soil surface complexity has a larger effect on food exploitation by ants than a change from grassland to shrubland. Ecol. Entomol. **43**, 379–388. (10.1111/een.12510)

[45] Wystrach A, Buehlmann C, Schwarz S, Cheng K, Graham P. 2020 Rapid aversive and memory trace learning during route navigation in desert ants. Curr. Biol. **30**, 1927–1933.(10.1016/j.cub.2020.02.082) 32275874

[46] Abràmoff MD, Magalhães PJ, Ram SJ. 2004 Image processing with ImageJ. Biophoton. Int. **11**, 36–42.

[47] Bega D, Samocha Y, Yitzhak N, Saar M, Subach A, Scharf I. 2019 The effect of maze complexity on maze-solving time in a desert ant. Behav. Processes **166**, 103893. (10.1016/j.beproc.2019.103893) 31252072

[48] McIntyre NE, Wiens JA. 1999 How does habitat patch size affect animal movement? An experiment with darkling beetles. Ecology **80**, 2261–2270. (10.1890/0012-9658(1999)080[2261:HDHPSA]2.0.CO;2)

[49] McIntyre NE, Wiens JA. 1999 Interactions between landscape structure and animal behavior: the roles of heterogeneously distributed resources and food deprivation on movement patterns. Landsc. Ecol. **14**, 437–447. (10.1023/A:1008074407036)

[50] Hake M, Ekman J. 1988 Finding and sharing depletable patches: when group foraging decreases intake rates. Ornis. Scand. **19**, 275. (10.2307/3676721)

[51] Rita H, Ranta E, Peuhkuri N. 1996 Competition in foraging groups. Oikos **76**, 583. (10.2307/3546353)

[52] Treherne JE, Foster WA. 1982 Group size and anti-predator strategies in a marine insect. Anim. Behav. **30**, 536–542. (10.1016/S0003-3472(82)80066-3)

[53] Lehtonen J, Jaatinen K. 2016 Safety in numbers: the dilution effect and other drivers of group life in the face of danger. Behav. Ecol. Sociobiol. **70**, 449–458. (10.1007/s00265-016-2075-5)

[54] Polyakov AY, Quinn TP, Myers KW, Berdahl AM. 2022 Group size affects predation risk and foraging success in Pacific salmon at sea. Sci. Adv. **8**, eabm7548. (10.1126/sciadv.abm7548) 35767621 PMC9242597

[55] Taylor K, Visvader A, Nowbahari E, Hollis KL. 2013 Precision rescue behavior in North American ants. Evol. Psychol. **11**, 665–677. (10.1177/147470491301100312)

[56] Lucas JR. 1985 Partial prey consumption by antlion larvae. Anim. Behav. **33**, 945–958. (10.1016/S0003-3472(85)80029-4)

[57] Pollard SD. 1989 Constraints affecting partial prey consumption by a crab spider, Diaea sp. indet. (Araneae: Thomisidae). Oecologia **81**, 392–396. (10.1007/BF00377089) 28311194

[58] Samu F. 1993 Wolf spider feeding strategies: optimality of prey consumption in Pardosa hortensis. Oecologia **94**, 139–145. (10.1007/BF00317315) 28313872

[59] Barkae ED, Scharf I, Ovadia O. 2017 Differential effects of variance in prey arrival on foraging success and growth rate of two pit‐building antlion species. J. Zool. **303**, 254–260. (10.1111/jzo.12487)

[60] Bolek S, Wolf H. 2015 Food searches and guiding structures in North African desert ants, Cataglyphis. J. Comp. Physiol. A **201**, 631–644. (10.1007/s00359-015-0985-8) PMC4439442 25663433

[61] Pfeffer SE, Bolek S, Wolf H, Wittlinger M. 2015 Nest and food search behaviour in desert ants, Cataglyphis: a critical comparison. Anim. Cogn. **18**, 885–894. (10.1007/s10071-015-0858-0) 25805650

[62] Bartal IBA, Decety J, Mason P. 2011 Empathy and pro-social behavior in rats. Science **334**, 1427–1430. (10.1126/science.1210789) 22158823 PMC3760221

[63] Collett TS, Dillmann E, Giger A, Wehner R. 1992 Visual landmarks and route following in desert ants. J. Comp. Physiol. A **170**, 435–442. (10.1007/BF00191460)

[64] Pembury Smith MQR, Ruxton GD. 2020 Camouflage in predators. Biol. Rev. **95**, 1325–1340. (10.1111/brv.12612) 32410297

[65] Shine R, Li-Xin S. 2002 Arboreal ambush site selection by pit-vipers Gloydius shedaoensis. Anim. Behav. **63**, 565–576. (10.1006/anbe.2001.1928)

[66] Heinrich B, Heinrich MJE. 1984 The pit-trapping foraging strategy of the ant lion, Myrmeleon immaculatus Degeer (Neuroptera: Myrmeleontidae). Behav. Ecol. Sociobiol. **14**, 151–160. (10.1007/BF00291906)

[67] Scharf I, Ovadia O. 2006 Factors influencing site abandonment and site selection in a sit-and-wait predator: a review of pit-building antlion larvae. J. Insect Behav. **19**, 197–218. (10.1007/s10905-006-9017-4)

[68] Bar A, Raveh-Pal E, Subach A, Foitzik S, Scharf I. 2024 Supplementary material from: Desert ants avoid ambush predator pits depending on position, visual landmarks, and trapped nestmates. FigShare (10.6084/m9.figshare.c.7576455) PMC11651900 39698156

